# How primary care can contribute to good mental health in adults*

**DOI:** 10.1080/17571472.2017.1410043

**Published:** 2017-12-13

**Authors:** Sunjai Gupta, Rachel Jenkins, John Spicer, Marina Marks, Nigel Mathers, Lise Hertel, Laura Calamos Nasir, Fiona Wright, Baljeet Ruprah-Shah, Brian Fisher, David Morris, Kurt C. Stange, Robert White, Gina Giotaki, Tony Burch, Catherine Millington-Sanders, Steve Thomas, Ricky Banarsee, Paul Thomas

**Affiliations:** a Department of Health Services and Population Research, Institute of Psychology, Psychiatry and Neuroscience, Kings College, London, UK; b Honorary Consultant Psychiatrist, South London and Maudsley NHS Trust, London, UK; c Epidemiology and Mental Health Policy, Kings College London, ETHICS, London, UK; d Primary Care Education, Health Education England, London, UK; e Educational Trust for Health Improvement through Cognitive Strategies (ETHICS), London, UK; f Primary Medical Care, University of Sheffield, Sheffield, UK; g Integrated Care University College London Hospitals NHS Foundation Trust, London, UK; h School of Nursing, University of North Carolina at Chapel Hill, Chapel Hill, USA; i Public Health Barking and Dagenham Council and Greater London Authority, London, UK; j Accomplish Consultancy, London, UK; k Health Empowerment Leverage Project, New NHS Alliance, London, UK; l Mental Health, Inclusion and Community, University of Central Lancashire, Preston, UK; m Family Medicine and Community Health, Epidemiology and Biostatistics, Oncology and Sociology, Center for Community Health Integration, Case Western Reserve University, Cleveland, OH, USA; n Primary Care Mental Health Services, West London Mental Health Trust, London, UK; o Dance Practices, Liverpool John Moores University, Liverpool, UK; p Health Education England North London, London, UK; q Marie Curie National Clinical End of Life Care Champion, London, UK; r Mental Health & Learning Disabilities NHS Sheffield Clinical Commissioning Group, Sheffield, UK; s Public Health England, London, UK; t London Journal of Primary Care, London, UK

**Keywords:** Mental health, primary care, collaboration, Accountable care organisations

## Abstract

The need for support for good mental health is enormous. General support for good mental health is needed for 100% of the population, and at all stages of life, from early childhood to end of life. Focused support is needed for the 17.6% of adults who have a mental disorder at any time, including those who also have a mental health problem amongst the 30% who report having a long-term condition of some kind. All sectors of society and all parts of the NHS need to play their part. Primary care cannot do this on its own. This paper describes how primary care practitioners can help stimulate such a grand alliance for health, by operating at four different levels – as individual practitioners, as organisations, as geographic clusters of organisations and as policy-makers.

**Why this matters to us**

London Journal of Primary Care (LJPC) publishes articles on the multi-dimensional aspects of primary care that make it so human and vibrant. It is a network of people who want to develop holistic, community-oriented integrated care and health promotion as a force for whole-society health. We hope that this paper will help general practice and more broadly primary care, to take a strong role in developing this.

**Key message**

Primary care can have an enhanced effect on the good mental health of the population by collaborating with others within local communities for health.

In 2015, LJPC contributed to a Think Tank, in partnership with the mental health charity ETHICS and the Royal College of General Practitioners (RCGP) to identify things that primary care can do to promote good mental health throughout the population [1]. This translated into national RCGP policy; an  LJPC paper describes its 12 recommendations for action (Figure 1) [2].

**Figure 1.**
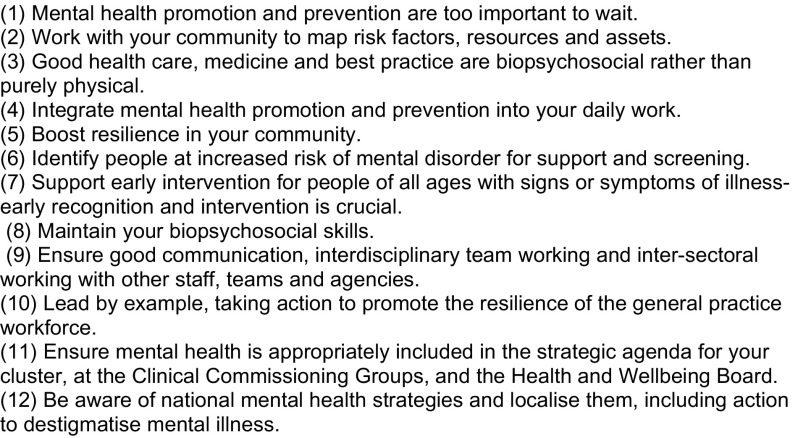
RCGP Recommendations as set out in Thomas et al. [2].

In this paper, we describe things that General Practices in the UK National Health (NHS) can do to translate these recommendations into practice. Different practices will want to do different things because of different health needs in different places, different skills of practice staff and different local services and resources. So the action areas are described as general aims. We invite you to apply them in ways that are relevant to your specific context.

The need is enormous. General support for good mental health is needed for 100% of the population, and at all stages of life, from early childhood to end of life. Focused support is needed for the 17.6% of adults who have a mental disorder at any time [1], including those who also have a mental health problem amongst the 30% who report having a long-term condition of some kind [3]. All sectors of society and all parts of the NHS need to play their part. Primary care cannot do this on its own.

Whatever the diagnosis and context, good mental health of adults is helped by good bio-psycho-social habits – good habits for physical fitness, for emotional centred-ness and for participation in initiatives that develop communities.

Making a significant improvement to the mental health of all citizens requires inter-sectoral collaboration at all levels, national, regional, district and local, in order to promote mental health. If we want to improve the mental health of the whole population, we need to develop a grand alliance for health and care. General practice, and more broadly primary care, is only one contributor, but well placed to have a coordinating role. Others are public health, mental health and social care services, voluntary care, schools, businesses and pretty much everyone else. To have effects which are more than the sum of the parts, these contributions need to be integrated, or at least aligned, in ways that help create and sustain environments that promote good mental health.

Aligning multiple efforts for good mental health is helped by a shared geographical locality. Working from a specific organisation (e.g. a general practice) helps those who belong to that organisation but it does not easily enable complex collaborations that have mutually enhancing effects. Recognition of the need for local collaboration for health dates from the 1978 World Health Organisation (WHO) international conference at Alma Ata [4], and reaffirmed in 2008 when the WHO Director-General explained why such localism is ‘more important than ever’ [5]. In the 2008 document, WHO specifically advocated community-based *hubs of coordination* to achieve comprehensive collaboration for health and care. More recently, the WHO’s Mental Health Action Plan 2013–2020 [6] aims to ‘implement strategies for promotion and prevention in mental health….’ and the ‘development of comprehensive community-based mental health and social care services’.

In England, the WHO vision for community-based coordinating hubs is being translated into policy through ‘New Care Models’ [7] (which bring together professionals to work collaboratively including GP, hospital, community and mental health services), and the ‘Primary Care Home’ [8] which aims to improve personalised and preventative care for the local population. The Primary Care Home provides care to a geographic locality of between 30,000 and 50,000. This is small enough to feel part of and large enough to have a political impact. In the horizontal direction, those involved develop a local community for health. In the vertical direction, they coordinate large numbers of care pathways. In future, there will be a shift in some activities from the Clinical Commissioning Group (CCG) to the Accountable Care Organisation (ACO), though CCGs will continue to be responsible and accountable for the delivery of their functions [9].

It is to leaders of community-based coordinating hubs that we target this paper. Figure 2 describes five different levels at which primary care practitioners can make a difference – as a citizen, as an individual practitioner, through the practice, through localities and through CCGs, Sustainability and Transformation Partnerships and ACOs. The first we leave to you to think about – how you personally contribute to your local community is your concern. We target this paper at the other four levels. We hope that this paper stimulates local discussion about how to make a collective impact that is more than any individual practitioner or discipline can achieve on its own.

**Figure 2.**
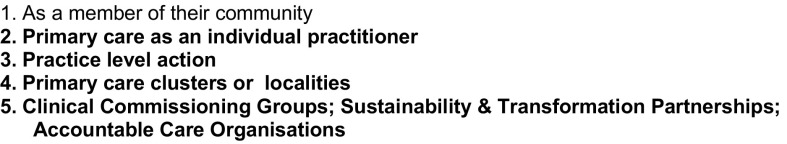
Levels at which primary care can take action [1,9,10].

## The case for action

• Each year, 70 million sick days are lost to mental disorders which are the leading cause of sickness absence in the UK [1]
• Only 43% of all those with mental health problems are in employment, in contrast to 74% of the general population and up to 65% of those with other health problems [11]
• Forty-four per cent of employment and support allowance benefit claimants report having a mental disorder as their primary diagnosis [1]
• It has been estimated that mental health-related job loss costs employers £82 million per year [12]
• Most people who commit suicide have seen their GP in the recent past [13]
• The life expectancy of adults with severe mental illness lags behind by an average of 10–20 years [14] and those with major depression and schizophrenia have a 40–60% elevated risk of dying prematurely [6]
• Medically unexplained symptoms are involved in 15–30% of all primary care consultations [14]
• About 1% of adults have autistic spectrum disorder but most are undetected [12]

These present a compelling case for primary care to contribute to a broad local collaboration to maintain good mental health of adults in the local population.

## General practice can

(1) Use initiatives like New Care Models and Primary Care Home to work with local general practices, public health, social care, mental health services, voluntary groups and others to *align plans for positive mental health* in the locality. This should encourage self-care, including use of digital technologies [15,16], and promote the ‘Five ways to mental wellbeing’ [17] (Connect, Be Active, Take Notice, Keep Learning, Give).
(2) Work in clusters of practices and their extended primary care teams to integrate mental health care into routine care, and plan with mental health and public health practitioners, social workers and voluntary groups. This makes it easier to persuade a range of other organisations and disciplines to contribute to an integrated strategy for good mental health. Shared planning in this way can improve:
  • Training for clinicians to be skilled at discerning ‘teachable moments’ when patients are ready to learn and change
  • Strategically planned access to psychological therapies
  • Employers taking responsibility for employees who have mental health problems
  • Inter-organisational collaboration that builds local communities for health [18]
(3) Write *live manuals,* tailored to local needs, to inform practitioner decisions, self-help and care pathways [19]. It is easy to forget simple things that can promote good mental health and improve mental illness. Web-based ‘live manuals’ that are regularly updated by multidisciplinary leadership teams and used every day by practitioners help avoid this problem and empower good practice when they:
  • Describe things that practitioners can do during consultations, including opportunistically assessing mental health of patients and using an empowering consulting style to discern ‘teachable moments’ when patients are open to learning and change
  • Signpost self-help options that help people know how common it is to feel anxious and depressed and how to improve their skills at reducing anxiety and avoiding projecting negative feelings onto others
  • Show how to refer into care pathways, supported by in-the-moment specialist advice and a facility to fast-track to specialist care those who need it
(4) Develop *multidisciplinary teams,* for the care of mentally unwell people, whose members are skilled at integrating mental health care into their everyday care [20,21].

Care plans for people with mental illness are a well-established and effective way to share care. Care planning teams should include more than mental health practitioners and include primary care clinicians, specialists, carers and many others. Each of these could contribute with links to resources that help develop a sense of positive connection with self and others, and enhance physical fitness, emotional centred-ness and participation in initiatives that develop social networks. Such resources could include participatory forms of art that build communities and enhance health and well-being of individuals and groups. Together, care planning teams could:

• Identify relapse at an early stage
• Share records and agree ‘Special Patient Notes’ to guide the actions of others who have occasional contact with patients (e.g. Out of Hours Services)
• Promote incremental improvements in lifestyle, well-being and relationships as the opportunities arise

(5) Highlight *wider determinants of mental illness*

Anxiety and depression commonly have a range of contributing factors including relationships difficulties, unresolved old conflict, grief, debt, alcohol misuse, lack of physical fitness and loss of purpose. These same factors worsen all mental illnesses. Highlighting them can help patients address them. Primary care practitioners can:

• Encourage patients to focus on controlling things they have the power to control and be less anxious about things they cannot control
• Refer into services for debt management, housing improvement, lifestyle improvement, domestic violence, anger management, alcohol misuse; and argue the need for continued funding of such services
• Support initiatives that build resilience, including helping people develop networks of trusted relationships which enable the community become more engaged and which build social capital

(6) Consider mental health needs of those who have *long*-*term conditions*

In England, 15.4 million people suffer from a long-term condition [3]. Many of those who have a long-term condition of one kind or another have Care Plans that include patient goals for self-care. The care planning process could highlight mental health issues by:

• Routinely asking about anxiety and depression
• Providing literature and courses to help people realise that it is common to have negative emotions to long-term illnesses and these can often be addressed through simple actions
• Reminding that medically unexplained symptoms and everyday complaints like backache and headache often have associated psychosocial problems that can be improved with purposeful actions

(7) *Signpost* ways for people to *self*-*care* and make *useful contributions to society*

Good mental health includes a positive sense of personal identity, including a coherent and positive sense of their life story in which the person is able to rise above the difficulties they encounter. Mental illness disturbs this coherence through internal things like anxiety and fear, and external things like weak relationships and contracted life options. A partnership of organisations in a locality can promote ways to reduce inner turbulence and increase external resilience. They can:

• Encourage everyone to make useful contributions to society, including paid and voluntary work that helps strengthen the local community, appreciate those around them and increase their webs of trusted relationships
• Encourage everyone to be skilled at being personally centred and able to listen positively to others, including things like mindfulness, yoga, meditation, music and gardening
• Encourage *healthy lifestyles* for everyone, including physical fitness, sport, dancing and weight control

**Figure 3.**
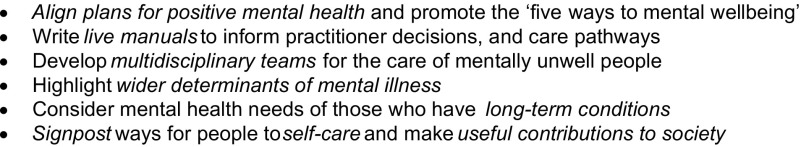
Examples of actions that primary care can undertake to improve mental health.

Some of these actions that primary care can undertake are summarised in Figure 3.

## Please contribute to LJPC discussions about mental health promotion in primary care

The 2014 Commonwealth Fund Report reveals that the UK enjoys some of the best health care in the world. When compared with 10 other developed countries, it ranked first overall, scored highest for quality, access and efficiency; and second equal for equity (along with Switzerland and behind Sweden). This is despite having the second lowest per capita spend (New Zealand was lower). However, in the category of ‘*healthy lives’,* it scored 10th – 2nd only to the USA for poor outcomes [22]. The category of ‘healthy lives’ includes: (1) mortality amenable to medical care, (2) infant mortality and (3) healthy life expectancy at age 60. These are all things that require many different organisations to align their efforts to promote health – public health, local authorities, schools, community groups, families and many others. We need to develop and evaluate models of complex collaborations for whole society health, including mental health.

Primary and community care practitioners don’t need the Commonwealth Fund to see the need for integrated working between different agencies for good mental health. Every time we see a patient who is anxious, or depressed, or lonely, or has difficult life circumstances, we recognise what’s needed – families, friends, neighbours and other social support; getting meals on tables, keeping active, getting out of doors. It is obvious, surely, that sometimes people need a variety of help to do these things, beyond specific therapies, and to take part in activities that put a smile on a face – music, dance, photos, chats, gardening….

LJPC intends to continue this debate about locality-based collaboration for positive mental health. We want to do this through a network of collaborating sites that support the evaluation of case studies of community-oriented integrated care. Their published papers (in LJPC and elsewhere) can fuel debate, including social media to gain deeper understanding of how modern-day primary care can support mentally healthy societies.

We want to publish evaluations of things like social prescribing and primary care navigators, Primary Care Homes and Vanguard Sites and other models that build local communities for health. This paper describes things that primary care can do to improve the mental health of adults. In time we hope to publish similar papers focused on children, parents and families, retirement, end of life care and refugees.

Please get involved.

## Governance

LJPC Board overviewed this work.
